# Tailoring the Microarchitectures of 3D Printed Bone-like Scaffolds for Tissue Engineering Applications

**DOI:** 10.3390/bioengineering10050567

**Published:** 2023-05-09

**Authors:** Eleonora Zenobi, Miriam Merco, Federico Mochi, Jacopo Ruspi, Raffaella Pecci, Rodolfo Marchese, Annalisa Convertino, Antonella Lisi, Costantino Del Gaudio, Mario Ledda

**Affiliations:** 1 Hypatia Research Consortium, Via del Politecnico snc, 00133 Rome, Italy; 2 E. Amaldi Foundation, Via del Politecnico snc, 00133 Rome, Italy; 3 Institute of Translational Pharmacology, National Research Council, Via Fosso del Cavaliere 100, 00133 Rome, Italy; 4 Biomedical Engineering, Department of Basic and Applied Sciences for Engineering, Sapienza University of Rome, Piazzale Aldo Moro, 00184 Rome, Italy; 5 National Centre for Innovative Technologies in Public Health, Istituto Superiore di Sanità, Viale Regina Elena, 00161 Rome, Italy; 6 Department of Clinical Pathology, Fatebenefratelli S. Peter Hospital, Via Cassia, 00189 Rome, Italy; 7 Institute for Microelectronics and Microsystems, National Research Council, Via Fosso del Cavaliere 100, 00133 Rome, Italy; 8 Italian Space Agency, Via del Politecnico snc, 00133 Rome, Italy

**Keywords:** 3D printing, biomimetic bone scaffolds, micro-CT, in vitro SAOS-2, hMSC evaluation, tissue engineering

## Abstract

Material extrusion (MEX), commonly referred to as fused deposition modeling (FDM) or fused filament fabrication (FFF), is a versatile and cost-effective technique to fabricate suitable scaffolds for tissue engineering. Driven by a computer-aided design input, specific patterns can be easily collected in an extremely reproducible and repeatable process. Referring to possible skeletal affections, 3D-printed scaffolds can support tissue regeneration of large bone defects with complex geometries, an open major clinical challenge. In this study, polylactic acid scaffolds were printed resembling trabecular bone microarchitecture in order to deal with morphologically biomimetic features to potentially enhance the biological outcome. Three models with different pore sizes (i.e., 500, 600, and 700 µm) were prepared and evaluated by means of micro-computed tomography. The biological assessment was carried out seeding SAOS-2 cells, a bone-like cell model, on the scaffolds, which showed excellent biocompatibility, bioactivity, and osteoinductivity. The model with larger pores, characterized by improved osteoconductive properties and protein adsorption rate, was further investigated as a potential platform for bone-tissue engineering, evaluating the paracrine activity of human mesenchymal stem cells. The reported findings demonstrate that the designed microarchitecture, better mimicking the natural bone extracellular matrix, favors a greater bioactivity and can be thus regarded as an interesting option for bone-tissue engineering.

## 1. Introduction

To date, additive manufacturing (AM) represents a sound solution to the needs of the research and enterprise sectors, being a layer-by-layer fabrication technology to reproduce specific three-dimensional patterns according to a computer-aided design (CAD) input. Starting from a properly designed stage, several fabrication methods can allow collection of geometrically complex samples, ensuring process repeatability and reproducibility. For this reason, AM has an essential role in product innovation and development thanks to its extreme flexibility, which is essential in the Fourth Industrial Revolution (now Industry 4.0) [1].

Focusing on the biomedical field, this technology can effectively pave the way for the definition of novel therapeutic protocols based on the development of ad hoc tissue-engineered scaffolds. Reasonably, each technique has unique characteristics and, depending on the properties that are requested for the 3D-printed scaffolds, it is therefore necessary to select the most appropriate one. The main AM technologies for tissue engineering applications include stereolithography (SLA), three-dimensional binder jetting printing (BJP), solution/slurry extrusion-based techniques (SETs), bioprinting (BP), and fused deposition modelling (FDM), which is comprised in the material extrusion additive manufacturing processes (ISO/ASTM, 52900:2021). The final result is strictly related to the fabrication methodology and to the processed materials as, e.g., STL deals with photopolymerizable solutions [2], also including ceramic particles [3]; BJP involves the controlled deposition of a binder material onto a powder bed [4,5]; SETs are a family of techniques in which a viscous solution is extruded from a vessel and the 3D structure is obtained with evaporation of the solvent [6,7]; BP is based on the fabrication of soft scaffolds, usually hydrogels, by printing biological materials [8]; and FDM realizes 3D structures by means of the extrusion of a polymeric filament from a heated nozzle and allows processing of custom-made filaments by blending different polymers and/or adding nanofillers [9,10,11]. Notwithstanding the actual AM potential to promptly reproduce tailored components, a number of limitations should be highlighted as well, depending on the final resolution, material(s) selection, and properties of the printed scaffolds, alongside the cost of the experimental setups. These points can play a pivotal role in the development of an effective biomimetic scaffold, capable of resembling the morphological arrangement of the natural extracellular matrix (ECM) of the tissue to be healed. A number of studies have addressed this issue, focusing on the role of the microarchitecture to be printed and the AM process to be suitably considered for this aim. Using SLA, it is possible to print trabecular-like biomimetic ceramic scaffolds by utilizing a high-loaded ceramic precursor resin. After the sintering step, the organic component totally degrades and a fully ceramic scaffold is obtained. This technique allows to deal with scaffolds which are fabricated at high resolution, but are generally too fragile [3,12]. To overcome this drawback, a polymer matrix can be a suitable option while an additive manufacturing process based on the material extrusion (MEX) can easily provide a valuable alternative. Exploiting its extreme repeatability [13,14] combined with the properties of the processed materials, it is possible to realize scaffolds with a morphological structure inspired by the natural structure of bone tissue, as presented in this study. Furthermore, dealing with ad hoc composites, tailored scaffolds with unique properties can be suitably printed for bone-tissue engineering applications [15].

In this regard, implementing the material extrusion technique, polylactic acid (PLA) was here processed to prepare three different scaffolds with a random and porous microarchitecture to provide a biomimetic environment similar to the trabecular morphology of human bone. Porosity plays an important role in allowing cells to migrate and reside within the scaffold. The size of the pores, their microarchitecture, morphology, and distribution have an effect not only on the ability of cells to colonize the scaffold, but also on their ability to proliferate and differentiate [16]. To assess the potential of the proposed approach for bone-tissue engineering, as a further improvement of an already-tested design process [17], the biological response of the 3D-printed scaffolds was evaluated by means of human osteosarcoma SAOS-2 cells, a well-characterized osteoblast-like model, and human mesenchymal stem cells (hMSCs). These stem cells are involved in normal human tissue renewal, wound healing, and in physiological response to injuries [18], and have shown repairing effects for the treatment of damaged tissues and degenerative diseases [19,20,21,22]. Notably, hMSCs secrete molecules that contribute to the recovery of the damage, acting in an indirect manner and activating endogenous cellular mediators of tissue regeneration [23,24,25]. In the early phase of tissue damage, the mesenchymal stem cells behave as an inflammation sensor, migrate to the injured site, and promote its regeneration through the production of immunoregulatory and pro-regenerative factors [26,27]. For this key role in the normal endogenous repair processes, MSCs have garnered great interest, although their survival and engraftment rates are low at the site of injury. Tailored investigations, including properly designed scaffolds such as the ones here presented, can pave the way for an effective and promising approach to address this limitation. The experimental assessment of a potential platform for bone-tissue engineering was therefore carried out to test the suitability of ad hoc novel biomimetic and bioinspired scaffolds.

## 2. Materials and Methods

### 2.1. Scaffold Design

For the design of the scaffolds, Meshmixer 3.5 software (v.2018, Autodesk, San Rafael, CA, USA) was used. Briefly, starting from a box-shaped solid with a representative size of a bone critical defect (10 × 10 × 3 mm^3^) [28], a three-dimensional random cluster of spheres was subtracted to obtain a porous pattern [29]. With the aim of dealing with three different bone models, the diameter (pore size) and the center-to-center distance (spacing) of the spheres were fixed at 500, 600, and 700 µm, in accordance with the range previously reported [30,31]. Subsequently, a mesh reduction was carried out for all the samples and the trabecular-like structure was obtained implementing a Delaunay triangulation [32]. The resulting models (labelled P1S1, P2S2, P3S3, respectively) were imported in IdeaMaker software (v 3.6.1, Raise 3D Inc., Irvine, CA, USA) to perform the slicing procedure and obtain the “GCode” file for the 3D printer. Models were sliced at 0.1 mm in order to reproduce the original CAD design as accurately as possible.

### 2.2. Scaffold Fabrication

The scaffolds and the control were fabricated by means of the Raise 3D N2 FFF printer (Raise 3D Inc., Irvine, CA, USA). A commercial polylactic acid (PLA) filament (FILOALFA, Turin, Italy; 1.75 mm diameter) was extruded at 205 °C through a 0.4 mm diameter nozzle; the bed temperature was set at 60 °C. All samples were fabricated with the same printing parameters. A PLA box-shaped control case (10 × 10 × 1.5 mm^3^) with no porosity was also 3D printed.

### 2.3. Morphological Characterization

For the morphological characterization of the scaffolds, a micro-computed tomography (micro-CT) evaluation was performed (SkyScan 1072; Bruker micro-CT, Kontich, Belgium). Scanning parameters were set as follows: 40 kV voltage, 248 μA current, 14.65 × 14.65 µm^2^ pixel dimension, corresponding to 20× magnification, and 180° sample rotation with a step of 0.45°. A 3D reconstruction of the scaffolds was carried out using NRecon software (Version 1.7.0; Bruker micro-CT, Kontich, Belgium), which was then analyzed by means of CT-Analyzer software (Version 1.16.9.0; Bruker micro-CT, Kontich, Belgium) to calculate the histomorphometric parameters [33].

### 2.4. Cell Culture

The human osteosarcoma SAOS-2 cell line was acquired from the American Type Culture Collection (ATCC, HTB-85 Rockvile, MD, USA). The cells were cultured in Dulbecco’s modified Eagle’s Medium, high-glucose (DMEM; Euroclone, Milan, Italy), with 10% heat-inactivated fetal bovine serum (FBS, Euroclone, Milan, Italy), 2 mM L-glutamine (Sigma, Darmstadt, Germany), 1.0 unit/mL penicillin (Sigma), and 1.0 mg/mL streptomycin (Sigma). The cells were grown in plastic dishes at 37 °C in a humidified incubator at 5% of CO_2_. After written informed consent, hMSCs were isolated from human term placenta in accordance with the Declaration of Helsinki, and the protocol was authorized by the Ethical Committee of the FBF S. Peter Hospital (committee’s reference number n. 64/2012/C.B June 2012). The hMSC isolation protocol is reported in Ledda et al. [34]. The hMSCs were cultured on plastic dishes in DMEM, 10% FBS, penicillin (100 U/ml) and streptomycin (100 μg/ml) (PBI International, Hastings, UK), EGF (10 ng/ml, ImmunoTools, Friesoythe, Germany) and β-mercaptoethanol (55 µM, Sigma-Aldrich, Darmstadt, Germany) and maintained at 37 °C in a humid atmosphere containing 5% CO_2_. For all tests, the scaffolds were soaked in ethanol 70% for 30 min and rinsed in phosphate buffered saline (PBS). SAOS-2 cells (3 × 10^4^ cells/cm^2^) and hMSCs (3 × 10^4^ cells/cm^2^) were grown for 4 days on the scaffold and on the PLA sample (CTR).

### 2.5. Cell Growth Analysis

SAOS-2 cell growth rate was studied using a cell proliferation colorimetric water-soluble tetrazolium salts oxidation test (Cell Proliferation Reagent WST-1; Roche Diagnostics, Monza, Italy). The SAOS-2 cells were seeded on the scaffolds and on the PLA sample (CTR) at a concentration of 3 × 10^4^ cells/cm^2^, grown for 4 days in a humidified incubator (37 °C, 5% CO_2_), and analyzed daily. At day 1, 2, 3, and 4, WST-1 chemical (1:10) was diluted in the medium of SAOS-2 cells and maintained for 2 h in incubator, the cell supernatants (100 µL) were transferred in a 96-well plate and the formazan dye was analyzed. The produced formazan dye was quantified with absorbance measurement at 450 nm with a scanning multiwell spectrophotometer (Biotrack II; Amersham Biosciences, Little Chalfont, UK).

### 2.6. Real-Time Quantitative RT-PCR Analysis

Total RNA was obtained from the SAOS-2 cells and hMSCs cultured on scaffold and on the PLA sample (CTR) for 4 days, using TRIzol Reagent (Invitrogen Waltham, USA). One microgram of total RNA was retrotranscribed using iScriptTM cDNA synthesis kit (Bio-Rad, Hercules, CA, USA). RT-qPCR was used for the quantification of studied mRNA with SsoAdvanced™ Universal SYBR^®^ Green Supermix (Bio-Rad Hercules, CA, USA) and Bio-Rad Real-Time PCR Detection Systems. The cDNA templates (0.5 µL) were run in triplicate in 20 µL of volume using with 250 nM of specific. The examined mRNA markers are listed in Table 1. Cycling parameters and qPCR protocol are reported in Ledda et al. [34]. The data were analyzed using the 2^−ΔΔCt^ equation proposed by Livak [35]. Each experiment was performed three times, using three samples for each scaffold model.

### 2.7. Scanning Electron Microscopy Analysis

Cell-scaffold constructs were fixed at day 4 with 4% paraformaldehyde and the dehydrated samples were covered with thin film of gold and then analyzed with ZEISS SIGMA 300 field emission scanning electron microscopy (SEM). The morphological analysis was carried out at an accelerating voltage of 5 kV by using the secondary electron (SE) detector.

### 2.8. Protein Adsorption

The adsorbed proteins were revealed with the Bradford method. The scaffolds were immersed in 1 mL of Fetal Bovine Serum (FBS) and incubated at 37 °C in a 24-well plate. The protein concentration in the FBS solution was determined with a standard protein assay kit (Bio-Rad, Hercules, CA, USA). The proteins adsorbed by the scaffolds were determined by subtracting the quantity of proteins left in the FBS solution after adsorption from their quantity in the control FBS solution (without sample) in identical experimental conditions. Three scaffolds were assessed for each group at each timepoint.

### 2.9. ELISA Assay

The hMSCs were seeded at a density of 3 × 10^4^ cells/cm^2^ on scaffolds and on the PLA sample (CTR). At 3 and 7 days, cell supernatants were isolated, centrifuged at 1200 rpm for 5 min and kept at −80 °C until use. ELISA development kit (PeproTech^®^ EC Ltd., London, UK) was used for quantification of Tumor Necrosis Factor-α (TNF-α) released by the cells grown on the scaffolds. A serial dilution of supernatant samples and human recombinant standards was prepared in 1xPBS/0.05% Tween-20/0.1% BSA (Sigma-Aldrich^®^, Darmstadt, Germany), and analyzed in a microplate. Interleukin binding was identified with biotin-avidin detection step, after chromogen 2,2′-azino-bis (3-ethylbenzothiazoline-6-sulphonic acid (Sigma-Aldrich^®^, Dorset, UK) treatment with ELISA reader at 405 nm.

### 2.10. Statistical Analysis

For qPCR and protein absorption analysis, each experiment was performed three times in triplicate (*n* = 9). The results are reported as mean ± SD and statistical analysis was carried out by means of one-way ANOVA test followed by the Student-Newman-Keuls post hoc test for all pair-wise comparisons. For qPCR analysis of paracrine factors, statistical analysis was carried out by means of the Student’s test to assess differences. Significant level was set at *p* < 0.05.

## 3. Results and Discussion

### 3.1. Scaffold Evaluation

Scaffold porosities for P1S1, P2S2 and P3S3, as calculated with the design software, were 43.58%, 49.46%, and 53.77%, while the specific surface (total surface/scaffold volume) were 5.53 mm^−1^, 10.73 mm^−1^, and 10.96 mm^−1^, respectively. Porosity was also experimentally assessed by means of a gravimetric method, being 37.3 ± 1.1%, 44.0 ± 2.7%, and 50.4 ± 0.3% for P1S1, P2S2 and P3S3, respectively.

**Figure 1:**
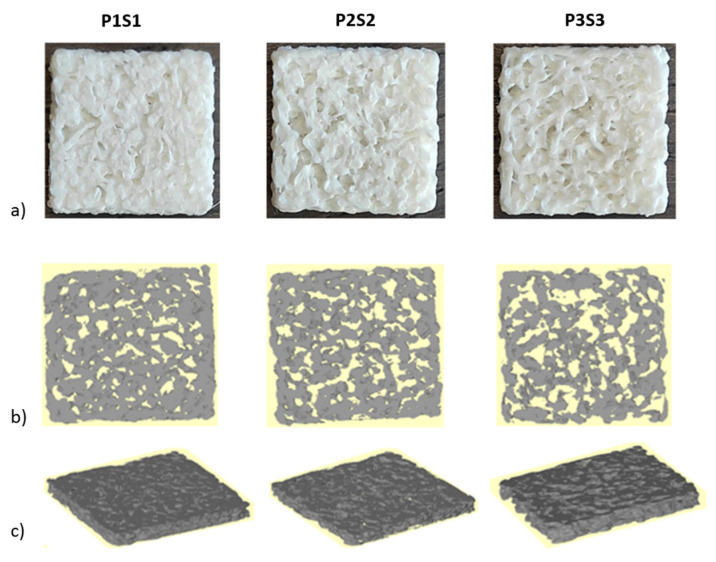
(**a**) Pictures of the 3D-printed PLA scaffolds; (**b**,**c**) Partial reconstruction of the internal architecture for each scaffold obtained with micro-CT.

Printed scaffolds were analyzed using the micro-CT to investigate the internal structure and compute the morphometric parameters. Pictures of the scaffolds and the reconstructed cross sections are shown in Figure 1.

Table 2 summarizes the most significative parameters obtained with micro-CT analysis for each set of the investigated samples, including a comparison with the analogue values of human proximal ulna, human calvarium, and human hemimandibular and hemimaxillae [36,37,38].

Percent bone volume inversely decreases with respect to pore size and separation. An increase in the specific surface (pivotal for cell adhesion and proliferation) can be highlighted while no significant change in trabecular thickness or separation is reported. This effect, as well as that of the discrepancy between the calculated and measured porosity and specific surface, can be related to the printer resolution limits and has already been extensively documented in the literature [39,40].

Considering the obtained porosity values, the P3S3 model is within the physiological range of the human ulna, hemimandibular, and hemimaxillae, and is very similar to the value of the human calvarium, while P1S1 is far from the physiological conditions [36,37,38]. Furthermore, it is well-known that trabecular bone porosity is strongly dependent on the site of interest, gender, and age of the subject [41,42,43]. It should be also underlined that the porosity of the three scaffolds is substantially open, and this occurrence allows to deal with a highly interconnected microstructure, which is a crucial requirement for support cell migration within the engineered construct.

Regarding trabecular thickness, all the specimens are close to the reference values; trabecular separation is different from all anatomical sites reported in Table 2, which is the morphometric parameter most affected by printer resolution.

### 3.2. Adhesion, Growth, and Differentiation of Osteoblast-like Cells on Biomimetic 3D-Printed Scaffolds

**Figure 2:**
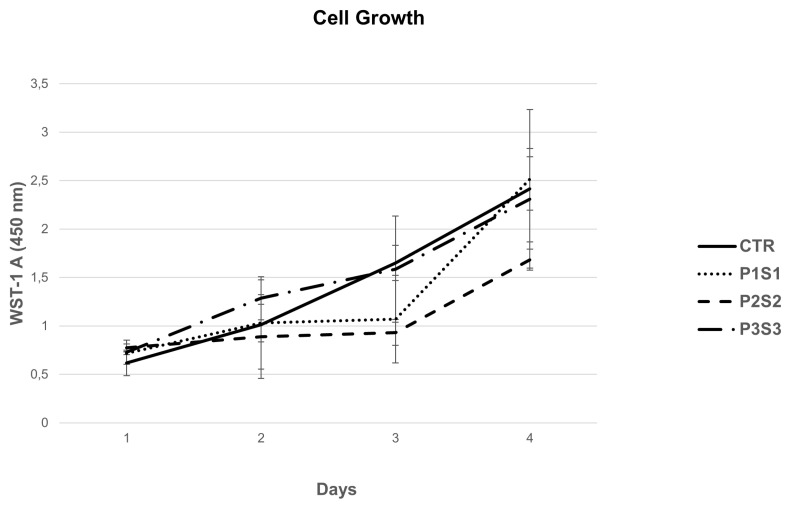
SAOS-2 growth analysis on P1S1, P2S2, P3S3, and PLA control (CTR) with WST-1 assay.

**Figure 3:**
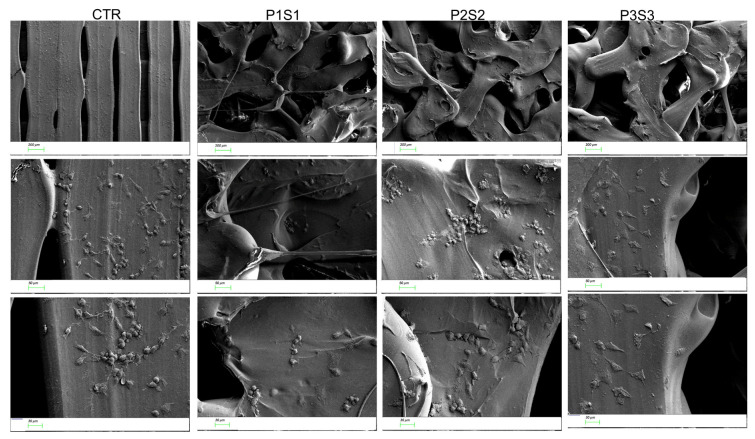
SEM micrographs of SAOS-2 seeded on the CTR, P1S1, P2S2, and P3S3 scaffolds at three different magnifications (magnification and scale bar: 50× and 200 µm upper panel, 200× and 50 µm middle panel, and 300× and 30 µm lower panel).

Human osteosarcoma cells, SAOS-2, represent a well-characterized bone model, thanks to their osteoblastic properties, including the ability to produce bone-like mineralized matrix and express mature osteocyte marker genes [44,45]. Moreover, this cell line is able to better mimic primary human osteoblast cell response in interaction with biomaterials, compared to other osteoblast-like cells [46]. In this regard, SAOS-2 cells have been selected for this study to assess scaffold biocompatibility and osteoconductive properties in vitro. Cells were seeded on PLA support (CTR) and on the three different models (P1S1, P2S2 and P3S3), their adhesion was investigated with SEM analysis, and growth capability was evaluated by means of the metabolic activity rate through the WST-1 colorimetric assay. The results show that the cells grown for four days on the three PLA scaffold models positively interacted with the polymeric substrates without any difference compared to the control case. In particular, the growth curve of the P3S3 scaffold showed an increased trend from day 0 to day 4, similar to CTR (Figure 2). Using SEM analysis (Figure 3), the influence of the PLA scaffolds on SAOS-2 adhesion and morphology was studied, also evaluating their migration. It can be noticed that the cells are well-attached on the scaffold substrates and, compared to the those grown on CTR, the morphology and size are significantly different. The cells on the three PLA scaffolds are more flattened and elongated compared to the CTR case, this effect is particularly evident for P3S3, the sample with a greater porosity, being an expected morphology in cells where the differentiation is ongoing, as later confirmed by the expression analysis of osteoblast mRNA markers. SEM micrographs confirmed a random microporosity architecture, different from the flat and well-ordered shape of the CTR.

**Figure 4:**
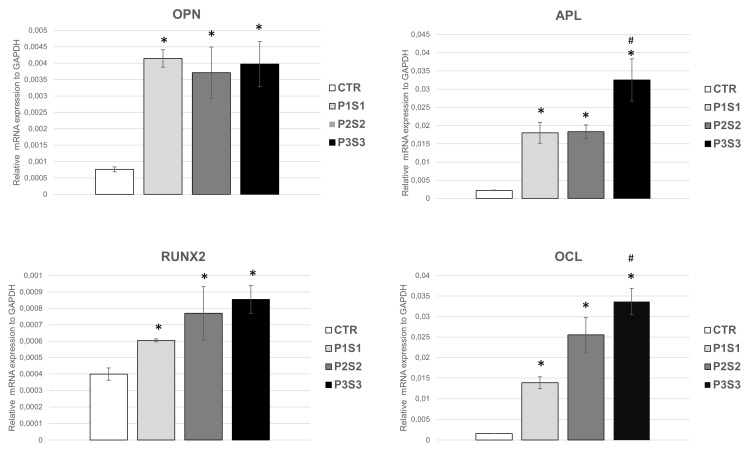
mRNA expression of the main osteogenic markers of SAOS-2 cell line. In particular OPN, APL, RUNX2 and OCL, evaluated by means of qRT-PCR analysis. * *p* < 0.05 with respect to CTR, # *p* < 0.05 with respect to P1S1 and P2S2.

Biocompatibility and osteoconductive properties of the 3D-printed scaffolds were further studied through the mRNA expression of osteoblast markers, i.e., osteopontin (OPN), alkaline phosphatase (APL), RUNX2, and osteocalcin (OCL), highly expressed in healthy growing SAOS-2 cells. Osteogenesis includes a series of events controlled by a combined gene expression cascade in which three sequential phases are identifiable: proliferation, ECM deposition and maturation, and mineralization of the bone ECM [47]. These phases are characterized by the transcription factor activation of specific genes related to the osteoblast phenotype, such as alkaline phosphatase, osteocalcin, osteopontin and RUNX-2. ALP is an early differentiation marker produced at the end of the proliferative stage and when ECM deposition and maturation occur. Both OPN, a key bone phosphorylated glycoprotein, and OCL, a highly conserved small molecule, are produced during the mineralization of the bone matrix, in the last phase of osteogenesis [48]. The expression of OPN, ALP, RUNX2, and OCL mRNA markers, studied with qRT-PCR assay, in SAOS-2 cells grown for 4 days on PLA scaffolds (Figure 4), revealed a statistically significant increase in the four markers for P1S1, P2S2, and P3S3 compared to the control sample. These findings highlight that the differentiation process is efficiently activated and is up-regulated, as shown by the higher levels of early and late bone differentiation markers expressed in all the three printed PLA models with respect to CTR. It is worth noting that the APL and OCL marker expression resulted statistically significant enhanced in P3S3 samples compared to the other two types of scaffolds. These results indicate a better osteoinduction capability, maybe due to microporosity which, for P3S3 scaffold, is more similar to those of bone physiological conditions. In fact, the bioinspired 3D microarchitectures and microporosity of this scaffold, mimicking natural bone extracellular matrix, favor bioactivity, and this can contribute to reproduce an osteoinductive microenvironment and stimulate osteogenic differentiation. To support this assumption, several studies report a significant role played by microporosity in enhancing the osteoinduction of 3D scaffolds [49], thanks also to an increase in the scaffold surface area, providing more protein adsorption sites which facilitate the interactions between constructs and cells, improving nutrient availability [50].

### 3.3. Protein Adsorption of 3D Printed Scaffolds

When biomaterials interact with a physiological system, the proteins in the medium are rapidly adsorbed on the surface and affect the subsequent cell interactions [50]. Hence, proteins are first adsorbed onto the scaffold surface, then cells adhere, and this process can affect the successive cell response [51]. An important consequence of protein adsorption is the creation of a biologically active surface, providing a favorable microenvironment that mediates key biological responses such as cell adhesion, viability, proliferation, and osteogenic induction. In addition, it has been reported that protein adsorption, as well as cell–material interaction, is correlated to microporosity that plays a key role in many biological processes [16] and affects the loading and release of specific molecules with regenerative potential. Key factors that strongly influence protein adsorption are the chemistry of the surface, the wettability, and the topography (e.g., roughness).

**Figure 5:**
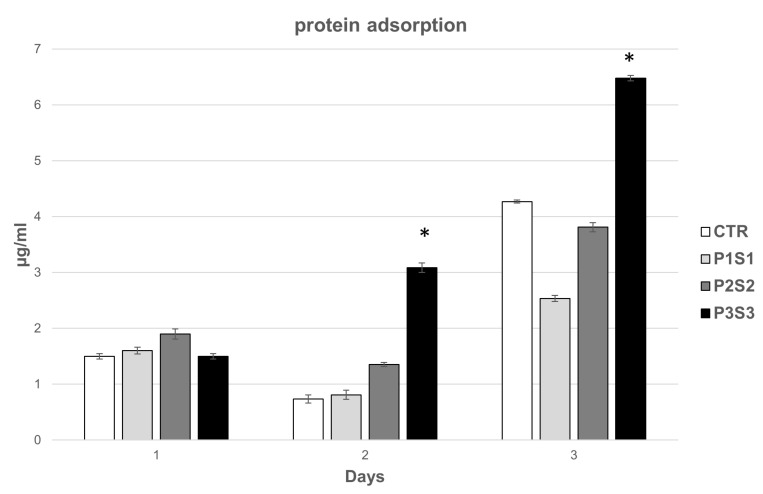
Protein absorption of the P1S1, P2S2, and P3S3 scaffolds compared to PLA control. * *p* < 0.05 with respect to CTR.

To explore scaffold bioactivity, the protein adsorption was quantified at different incubation time points (1, 2, and 3 days) and compared to CTR, mainly increasing for P3S3 from 1 to 3 days (Figure 5). The obtained data indicate that the microarchitecture of this scaffold, characterized by a higher porosity and a related specific surface area, enhances protein adsorption capacity which in turn can stimulate the osteogenic-related cell functions, such as attachment, proliferation and osteogenic differentiation [49].

### 3.4. Human Mesenchymal Stromal Cell Interaction with 3D-Printed Biomimetic Scaffolds (P3S3)

Multipotent mesenchymal stem cells (MSCs) are a promising option for tissue engineering and cell-based therapies thanks to their self-renewal capability and differentiation potential. These adult stem cells were isolated and analyzed by Friedenstein in 1971 [52] and are able to differentiate into different mesoderm lineages, such as osteocytes, chondrocytes, and adipocytes, as well as ectodermic and endodermic cell lineages [53,54,55,56,57]. Furthermore, hMSCs have raised great attention in the scientific community for clinical applications, as they have some features, such as homing to injured tissue sites, immune-tropic functions, and paracrine signalling, which open up the possibility of their use for tissue regeneration or immunological/inflammatory disorders treatment.

Their osteogenic differentiation ability and the immunomodulatory, anti-inflammatory and anti-apoptotic properties make hMSCs an optimal choice for cell therapy in the treatment of bone-tissue diseases [58,59,60], with the paracrine effect recognized as the main mechanism by which these cells affect tissue regeneration, with relevant therapeutic implications.

The beneficial effects of mesenchymal stem cells in tissue regeneration mostly depend on their capability to produce a large variety of soluble bioactive trophic and anti-inflammatory factors. This occurrence promotes several key biological activities for repairing damaged tissue. This paracrine activity has a very high potential also for bone regeneration mediated by the secretion of bioactive factors such as Insulin-like Growth Factor-1 (IGF-1), Transforming Growth Factor (TGF-ß1), Vascular-Endothelial Growth Factor (VEGF), Hepatocyte Growth Factor (HGF), and Bone Morphogenetic Protein (BMP-1) [61,62,63]. These cytokines regulate several events of osteogenesis, angiogenesis, cell migration and proliferation [62]. In particular, VEGF, the main regulator of angiogenesis, promotes survival and differentiation of endothelial cells and supports osteogenesis [64]. TGF-β1 contributes to bone formation by osteoprogenitor cell recruiting, proliferation, and differentiation into osteocytes [65]. Moreover, it is expressed during the development of the alveolar bone, periodontal ligament, and cementum [66]. HGF supports engraftment of hMSCs into the target injured tissue, enhancing their motility, propagation, and sustainability [67]. Cyclooxygenase 2 (COX-2) is a key enzyme involved in the synthesis of immunoregulatory factor PGE-2, strongly related to MSC immunomodulatory ability, and indoleamine 2,3-dioxygenase 1 (IDO), an anti-inflammatory factor, plays an important role in T-reg stimulation and in suppressing T-cell proliferation [68]. hMSCs can be considered a suitable option in regenerative medicine thanks to their paracrine effect, but their low cell engraftment and survival within the target tissue continue to be key limitations to the successful application of cell-based therapy in clinics. In fact, a direct and local cell injection often entails reduced cell survival and low engraftment due to the severe conditions and hostile environment at the damaged tissue area. To improve cell survival and engraftment after transplant, stem cells can be combined with scaffolds. As reported above, the P3S3 sample showed a higher protein absorption compared to the others investigated in this study (i.e., P1S1 and P2S2), which could be again related to the microporous architecture, mimicking the bone structure. This biomimetic characteristic could improve the stem cells’ survival and engraftment in the target tissue, making P3S3 a novel potential platform for bone-tissue engineering applications.

**Figure 6:**
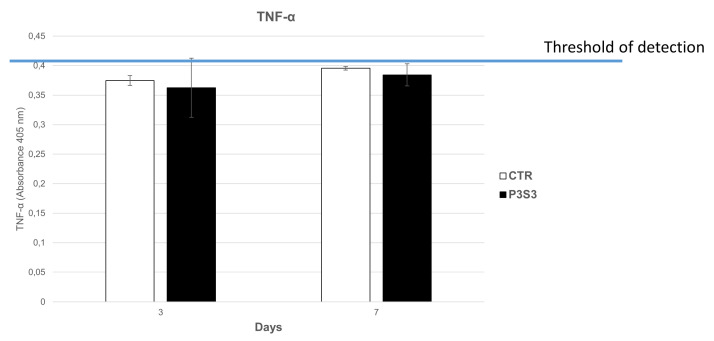
TNF-α, levels measured in the supernatant of hMSCs grown on P3S3 scaffold and in control samples at 3 and 7 days.

**Figure 7:**
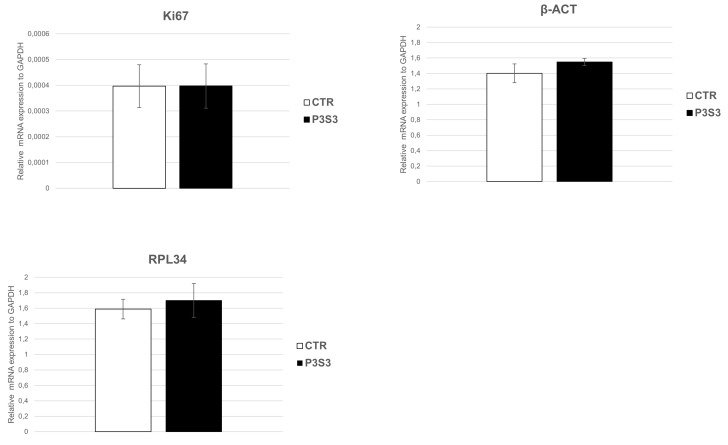
qRT-PCR analysis of hMSC key gene expression. The genes β-ACT, Ki67, and RPL34 were assessed in hMSCs cells grown on P3S3 scaffolds and compared to PLA substrate (CTR).

To verify this hypothesis, the hMSC–biomaterial interaction was assessed in terms of biocompatibility and bioactivity. The first one was evaluated by studying the pro-inflammatory TNF-α levels secreted by hMSCs grown on P3S3, and the expression of three constitutive key genes highly expressed in hMSCs: β-actin (β-ACT), which plays an important role in cell motility, structure, and integrity; Ki67, involved in cellular proliferation; and RPL34, a key ribosomal protein for cell translation process. The analysis of Tumor Necrosis Factor (TNF-α) protein levels, with ELISA assay, did not show detectable amounts of this pro-inflammatory factor in the cell medium of hMSC grown on the P3S3 scaffold for 3 and 5 days (Figure 6). Moreover, RT-qPCR assay showed no significant differences in gene expression between hMSCs grown on P3S3 and the control case (Figure 7). The high levels of mRNA expression of these three important constitutive genes maintained in hMSCs, and the absence of an inflammatory reaction after cell interaction with the substrate, confirm the P3S3 scaffold biocompatibility, already demonstrated using SAOS-2 bone-like cells.

Therefore, in this study, the expression of the bioactive factors, pivotal for the pro-regenerative potential of hMSCs, was investigated to evaluate the capability of P3S3 to support the hMSC paracrine activity that has been proposed as the principal mechanism of hMSC therapeutic effects [69].

**Figure 8:**
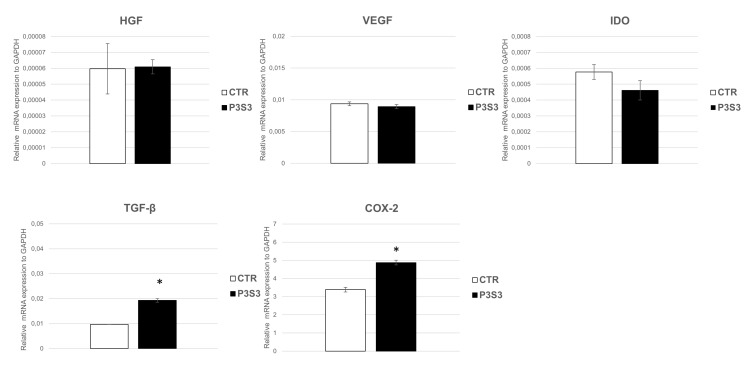
RT-qPCR analysis of bioactive (VEGF, HGF, IDO, TGF-β, COX-2) genes in hMSCs seeded on P3S3 scaffold compared to control. * *p* < 0.05 with respect to CTR.

Using qPCR analysis (Figure 8), a mRNA expression of VEGF, HGF, and IDO was highlighted, together with a statistically significant up-regulation of TGF-β and COX-2. This could have positive implications for bone regeneration, as TGF-β is expressed during bone development and repair and its local availability in the wound might cooperate for faster and successful bone healing [70]; while, COX-2 is a key enzyme involved in the MSC immunomodulatory ability. These results indicate that P3S3 scaffolds support hMSC paracrine signaling, paving the way for the use of this substrate as a new potential regenerative platform for bone-tissue repair. It can play a key role in the complex cascade of biochemical events controlled by many cytokines and growth factors, providing local signals for migration, proliferation, and differentiation of osteoprogenitor cells, and revascularization and production of extracellular matrix [70].

## 4. Conclusions

The study presented here aimed to reproduce ad hoc microarchitectures for bone-tissue engineering using a biomimetic approach. Polylactic acid scaffolds were designed 3D printed including different morphological features to provide a bone-like microstructure. Comparing human trabecular bone from different anatomical sites, i.e., skull, mandible, ulna, and maxilla, with the results from micro-CT investigation, P3S3 resulted in the scaffold most similar to the physiological case, according to porosity, specific surface, and trabecular thickness values. SEM investigation and biological assays confirmed that P3S3 scaffold was a promising bone model, supporting adhesion, proliferation, and osteogenic differentiation of the SAOS-2 cell line. In addition, the porosity degree promoted protein adsorption, suggesting a potentialbioactivity of the construct.

P3S3 scaffold represented an efficient means for the growth of mesenchymal cell that maintain their typically paracrine activity, as indicated by the mRNA expression of the main hMSC genes involved in this process. The adhesion and proliferation of hMSCs on the proposed scaffolds, following further analysis, could control and mitigate the inflammation process and optimize the repair of tissue injury, especially in bone-tissue engineering.

As a next step, the biomimetic features of the substrates could be improved by adding ad hoc fillers, such as ECM components of the bone (e.g., collagen and calcium phosphates). Moreover, as the 3D-printing technique here considered is capable of providing tailored models for biomedical investigation, the presented approach can be suitably planned to develop ad hoc innovative solutions in regenerative medicine field, allowing the printing of bone-like scaffolds in physio-pathological conditions and for specific anatomical sites.

## Figures and Tables

**Table 1:** Sequence of primers used for qRT-PCR.

Target Gene	Primer Sequence	Annealing Temperature (°C)
β-ACT	5′-gctcctcctgagcgcaag-3′ 5′catctgctggaaggtggaca-3′	60
OPN	5′-gtgtggtttatggactgagg-3′ 5′-acggggatggccttgtatg-3′	60
Ki67	5′-tgaacaaaaggcaaagaagac-3′ 5′-gagctttccctattattatggt-3′	60
RPL34	5′-gaaacatgtcagcagggcc-3′ 5′-tgactctgtgcttgtgcctt-3′	60
RUNX2	5′-catcatctctgccccctct-3′ 5′-actcttgcctcgtccactc-3′	60
ALP	5′-caatgagggcaccgtggg-3′ 5′-tcgtggtggtcacaatgcc-3′	60
OCL	5′-cagcgaggtagtgaagag-3′ 5′-gaaagccgatgtggtcagc-3′	60
GAPDH	5′-catcatctctgccccctct-3′ 5′-caaagttgtcatggatgacct-3′	60
VEGF	5′-cttgggtgcattggagcct-3′ 5′-ctgcgctgatagacatccat-3′	60
HGF	5′-caatagcatgtcaagtggag-3′ 5′-ctgtgttcgtgtggtatcat-3′	60
TGF β1	5′-tcaagttaaaagtggagcagc-3′ 5′-actccggtgacatcaaaaga-3′	60
IDO	5′-tgctaaaggcgctgttggaa-3′ 5′-tacaccagaccgtctgatag-3′	60

**Table 2:** Morphometric parameters of the 3D-printed scaffolds with a comparison from the literature data.

	P1S1	P2S2	P3S3	Human Proximal Ulna [38]	Human Calvarium [36]	Human Hemimandibular and Hemimaxillae [37]
Percent Bone Volume (%)	71.99	62.78	55.26	43.70 ± 22.40	46.70	37.29 ± 17.96
Total Porosity (%)	28.01	37.22	44.74	56.30 ± 22.40	53.30	62.71 ± 17.96
Open Porosity (%)	27.87	37.15	44.67			
Trabecular Thickness (mm)	0.31	0.28	0.26	0.40 ± 0.09	0.27	0.30 ± 0.08
Trabecular Separation (mm)	0.19	0.24	0.28	0.63 ± 0.22	0.59	0.59 ± 0.22
Specific Surface (mm^−1^)	10.22	12.57	14.30	NA	NA	12.79 ± 4.60
